# Repeated exposure to odors induces affective habituation of perception and sniffing

**DOI:** 10.3389/fnbeh.2014.00119

**Published:** 2014-04-10

**Authors:** Camille Ferdenzi, Johan Poncelet, Catherine Rouby, Moustafa Bensafi

**Affiliations:** Centre National de la Recherche Scientifique UMR5292, INSERM U1028, Centre de Recherche en Neurosciences de Lyon, Université Claude Bernard Lyon 1Lyon, France

**Keywords:** pleasantness, smell, repeated exposure, sniff, habituation

## Abstract

Olfactory perception, and especially hedonic evaluation of odors, is highly flexible, but some mechanisms involved in this flexibility remain to be elucidated. In the present study we aimed at better understanding how repeated exposure to odors can affect their pleasantness. We tested the hypothesis of an affective habituation to the stimuli, namely a decrease of emotional intensity over repetitions. More specifically, we tested whether this effect is subject to inter-individual variability and whether it can also be observed at the olfactomotor level. Twenty-six participants took part in the experiment during which they had to smell two odorants, anise and chocolate, presented 20 times each. On each trial, sniff duration and volume were recorded and paired with ratings of odor pleasantness and intensity. For each smell, we distinguished between “likers” and “dislikers,” namely individuals giving positive and negative initial hedonic evaluations. Results showed a significant decrease in pleasantness with time when the odor was initially pleasant (“likers”), while unpleasantness remained stable or slightly decreased when the odor was initially unpleasant (“dislikers”). This deviation toward neutrality was interpreted as affective habituation. This effect was all the more robust as it was observed for both odors and corroborated by sniffing, an objective measurement of odor pleasantness. Affective habituation to odors can be interpreted as an adaptive response to stimuli that prove over time to be devoid of positive or negative outcome on the organism. This study contributes to a better understanding of how olfactory preferences are shaped through exposure, depending on the individual's own initial perception of the odor.

## Introduction

Olfactory perception is known to be highly flexible as a function of perceiver's age, sex, or motivation state, of the context where the odor is perceived or of the characteristic of the odorant itself like its structure or its concentration. Another prominent factor of variations in odor perception is repeated exposure, which is able to improve olfactory detection thresholds (Stevens and O'Connell, 1995; Dalton et al., 2002) and can even boost olfactory sensitivity in seemingly anosmic participants (Wysocki et al., 1989; Mainland et al., 2002). Some studies also investigated the effect of exposure on discrimination abilities. There is now clear evidence that unreinforced exposure to odors can improve discrimination between odorants in humans (Rabin, 1988; Jehl et al., 1995). In line with this, it has been shown that exposure to odor mixtures can alter the perceived quality of the individual components (Stevenson, 2001). For instance, exposure to wine or beer through personal experience or through controlled training improves the ability to discriminate between different wines or beers (Owen and Machamer, 1979; Peron and Allen, 1988; Melcher and Schooler, 1996).

Although these studies show that exposure improves odor perception through differentiation of stimulus features, dimensions, or categories, how repeated exposure to odors affects one of the most prominent dimension of olfaction, namely pleasantness, remains understudied. What happens when we are exposed to the same odorant repeatedly? Do we like it more, or on the contrary do we like it less, or does liking remain stable overtime? A pioneer work in the field conducted by Cain and Johnson (1978) showed that mere presentation of a given odor significantly changed its hedonic value. More specifically, repeated presentation of a pleasant odor (citral) led to a decreased pleasantness whereas repeated presentation of an unpleasant odor (isobutyric acid) led to a decreased unpleasantness. In others words, repeated exposure shifted odor pleasantness ratings toward neutrality, a phenomenon called by Cain and Johnson “affective habituation.” However, to the best of our knowledge, non-verbal correlates of self-reported decrease in pleasantness (for pleasant odors) and unpleasantness (for unpleasant ones), such as psychophysiological responses, remain very scarcely investigated in the olfactory domain (but see evoked potentials for the unpleasant pole in Croy et al., 2013). Such a physiological indicator would be of particular interest, because it would strengthen the notion that affective habituation phenomenon is not due to experimental demand or even to a change in the use of the subjective scale over time.

Non-verbal measures of odor hedonics include autonomous (Alaoui-Ismaili et al., 1997; Bensafi et al., 2002a), or motor responses as reflected by reaction time studies (Bensafi et al., 2002b; Jacob and Wang, 2006; Boesveldt et al., 2010) and by sniffing responses to odors (Bensafi et al., 2003, 2007). Indeed, research in animals and humans has shown that sniffing behavior, i.e., the motor component of olfaction, is of considerable importance in odor perception. Sniffing is driven by stimulus attributes such as odor concentration (Laing, 1983; Frank et al., 2003; Johnson et al., 2003), and induces by itself activation in human primary olfactory cortex (Sobel et al., 1998). Furthermore, there is psychophysiological evidence that sniffing is modulated by subjective pleasantness of an odor: sniff duration and sniff volume increase when pleasant odors are sampled compared to unpleasant ones (Frank et al., 2003; Mainland and Sobel, 2006; Bensafi et al., 2007, 2003). Moreover, even when participants are asked to maintain their sniff for a specific duration irrespective of odor content, they sniff pleasant odors stronger and for a longer time (Bensafi et al., 2007). Thus, measuring sniffing patterns has two main advantages in studies on odor hedonics. First, it allows testing whether modulations in pleasantness are consistent with modulations in physiological/motor response. Second, this measure appears less vulnerable than verbal ratings to modulation by explicit or voluntary strategies, which makes it a more objective measure of hedonic responses. The main aim of the present study was therefore to examine whether affective habituation is not only observed at the self-reported level but also reflected at the psychophysiological level, by modulating sniffing responses to pleasant and unpleasant odors.

One striking particularity of odor hedonic responses is their variation between individuals: whereas affective evaluation of a given odor is positive for some individuals, the same smell may be considered unpleasant by others. For example, Doty (1975) emphasized the “large differences between observers in regard to the assessment of odorant hedonicity” (p. 495) based on 10 odorants, and noted for example that benzaldehyde had a bimodal distribution with half of the participants describing it as unpleasant and the other half as pleasant. In the same line, Bensafi et al. (2012) showed that two CO_2_-odor mixtures received varied hedonic ratings from one participant to another, and revealed differential activations in the brain according to whether the stimulation was perceived as pleasant or unpleasant. Moreover, Lundström et al. (2006) evidenced variability between individuals as regards pleasantness of the smell of androstenone, going from unpleasant to neutral. These hedonic rating differences were accompanied by distinctive verbal descriptions and neural responses in olfactory evoked potentials: Individuals who gave the lowest pleasantness ratings described the smell as “sweaty” and “urinous” and showed larger P3 amplitudes than individuals who gave higher pleasantness ratings and who described the smell with non-body descriptors (“smoky,” “fresh,” “sweet,” and “chemical”). In accordance with this finding, Keller et al. (2007) showed that variation of olfactory receptor expression accounted for a significant part of olfactory perceptual differences, especially between likers and dislikers of androstenone. Neuroimaging studies also shed light on these inter-individual differences in hedonic ratings of smells. In an fMRI study, Rolls and McCabe (2007) showed that chocolate cravers rated this flavor as more pleasant than non-cravers, and that an increasing level of pleasantness was associated with an enhanced activity in the pregenual cingulate cortex, the medial orbitofrontal cortex, and the dorsolateral prefrontal cortex. Altogether, these findings suggest that in olfaction studies, and especially in these dealing with pleasantness, it is of the utmost importance to take into account inter-individual differences because they have significant implications at the peripheral and central levels of olfactory processing.

Therefore, the secondary aim of the present study was to investigate inter-individual variability of the effect of repeated exposure on perceptual ratings and sniffing activity. To this end, participants were exposed to odors for which a previous study revealed large hedonic variability between raters (anise and chocolate; Barkat et al., 2008). In our study, participants were classified as “likers” or “dislikers” for each particular smell based on their initial hedonic ratings. They were then exposed 20 times to each odorant while hedonic ratings and sniffing behavior were recorded. We hypothesized that: (1) olfactory repeated exposure should decrease odor pleasantness in “likers” and odor unpleasantness in “dislikers,” (2) such affective habituation should be accompanied by changes in sniff parameters, namely decreased sniff volume and duration in “likers” and increased sniff volume and duration in “dislikers.”

## Materials and methods

### Participants

Twenty-six young adults (mean age ± s.e.m: 21.5 ± 0.46, range 19–29; 18 women and 8 men) attending the Claude Bernard University of Lyon (France) participated in the experiment. The experimental procedure was explained in great detail to the participants, who provided written consent prior to participation. The study was conducted according to the Declaration of Helsinki and was approved by the local ethical committee. Based on participant's reports, exclusion criteria were: abnormal olfaction, history of neurological disease or injury, or history of nasal insult (broken nose or surgery).

### Odorants and olfactometry

**Figure 1:**
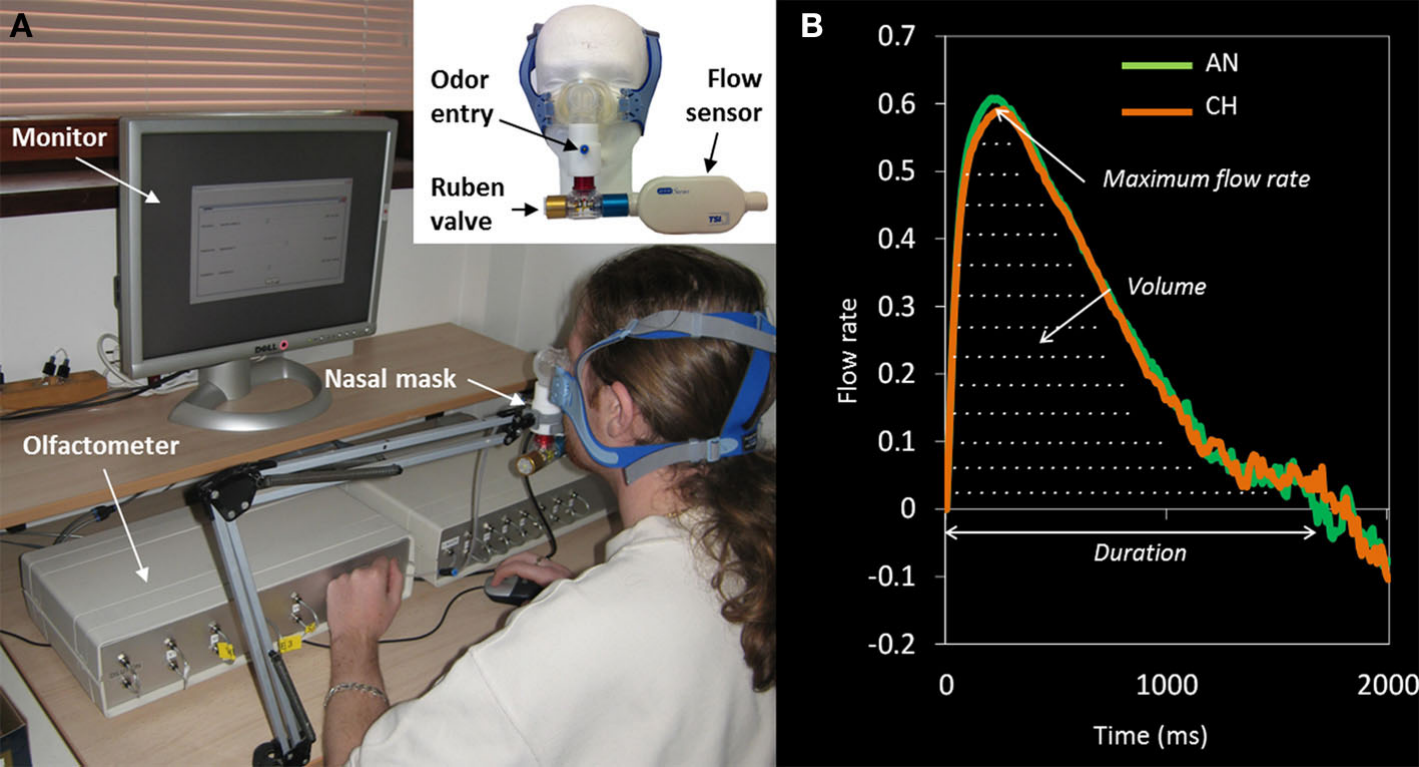
**(A)** Experimental device ensuring odor delivery and sniff recording. Nasal respiration was monitored with a flow sensor connected both to the subject's nose via a nasal mask and to the olfactometer. The nasal inspiration, detected by the flow sensor, triggers the sending of the odorant by the olfactometer during the requested duration to the subject's nose. To clean the mask chamber between stimulations, and to decrease the risk of odor contamination, the mask was connected to a Ruben valve (Intersurgical^®^, 7562700, 22F-22M/15F, UK) so that the odorized air contained in the chamber was sent out on each expiration. **(B)** Grand average of the sniffs for the odors of anise (AN) and chocolate (CH) across all trials and all participants, showing the maximum flow rate, duration, and volume.

Based on the results of a hedonic ranking task involving 8 odorants in a previous study (Barkat et al., 2008), two odorants, anise and chocolate (Euracli, France), were chosen because (1) they received a medium mean rank, and (2) they exhibited a large inter-individual variability. Odorants were diluted in mineral oil (10%) and presented to both nostrils via a nasal mask (Figure 1A). They were presented 20 times each in a random order, with an inter-stimulus interval of 30 s and duration of 3 s. Stimulations were delivered via a computer-controlled air-dilution olfactometer whereby odorants were diffused synchronously with the beginning of participant's inspiration (respiration was recorded continuously during the study).

The general principle of the olfactometer is to mix two airflows (odorized and pure air) to deliver a constant odorized or non-odorized airflow to the participant's nose. Pure air is sent by a compressor and cleaned by an activated carbon filter before being carried to the olfactometer input line (6 mm diameter, 5 m length tube). A manometer allows selecting the air input pressure. Then, air enters two channels: (1) a channel where it works as air carrier, and (2) an “odor” channel (one per odorant). For each odorant, a glass tube is set with polypropylene marbles where the odor is adsorbed. At the exit of each channel, an electric valve is programmed to be closed or open so that the odorant is pushed into the airflow for a given duration and pressure. The output odorous air is led by a 4 mm tube (20 cm length) into the nasal mask.

The experimental room was well-ventilated and included two areas, one for the experimenter and one for the participant. The experimenter area contained the computer controlling the olfactometer and two control screens showing the processing of the olfactometer and the answers the participant was giving on his/her own screen. The participant's area included the olfactometer output, as well as a screen and a mouse allowing them to read the instructions and give their ratings after each olfactory stimulation.

### Procedure

After providing written informed consent and reading instructions, participants were taken into the testing room. At this point, the experimenter fitted the sniffing equipment to the participants. Sniffing was recorded using an airflow sensor (TSL^®^, 4000 series, Model 40211, USA) connected to the nasal mask delivering odors to both nostrils. Sniffing signal was amplified and digitally recorded at 100 Hz using Python software^®^.

Upon installation of the nasal mask, the experiment started. Each trial was timed, and cued by the computer-generated visual instructions “please prepare to smell,” displayed for 3 s and announcing odor delivery. Once the instruction disappeared, participants were to sniff, which enabled the airflow sensor to detect the beginning of subject's inspiration and trigger odor delivery via the olfactometer. Following each odor presentation, participants rated stimulus pleasantness and intensity on an on-screen visual analog scale: the left end of the scale was labeled “extremely unpleasant” or “no stimulus perceived” (0), and the right end “extremely pleasant” or “extremely strong” (100). Instructions, odor presentation and sniffing recordings were all time-locked through one central computer.

### Data analysis

For each participant, we recorded intensity and pleasantness ratings (0–100) and sniff parameters on 20 occasions per odor (T1 to T20, for anise and chocolate). Sniffs (see Figure 1B) were pre-processed by removing baseline offsets and aligned in time by setting the point where the sniff entered the inspiratory phase as time zero. Sniff maximum flow rate, duration, and volume (see Figure 1B) were calculated for the first sniff of every trial, for every participant. Before analyzing how the ratings and sniff parameters changed with repeated exposure, outliers defined as values exceeding three standard deviations from the participant's mean were removed (0.65% of the trials). Then, analyses of the time-related changes in ratings and sniff parameters were performed (1) at the group level, by comparing time-related changes of “likers” (participants giving the highest pleasantness scores) and “dislikers” (participants giving the lowest pleasantness scores), and (2) at the individual level, by correlating each participant's initial pleasantness rating at T1 with the time-related changes across the trials T1 to T20. Time-related changes in hedonic and intensity ratings, sniff maximum flow rate, duration, and volume were represented by the slope of each variable as a function of trial number (1 to 20). A positive slope and a negative slope, respectively correspond to an increase and a decrease of the measured variable over time. For the group analysis, slopes were computed on average scores for “likers” and “dislikers” on each trial T1 to T20. The significance of the increases/decreases was assessed by using linear regressions with trial number as predictive variable. For the individual level analysis, slopes were computed for each participant individually. Correlation between individual pleasantness rating at T1 and the slopes of pleasantness, intensity and sniff parameters were investigated using Spearman rank correlation coefficient. Here, we expect “likers” to exhibit negative slopes and “dislikers” to display positive slopes in both perceptual and sniffing variables. This should be confirmed at the individual level by negative correlations between individual hedonic scores at T1 and individual slopes.

## Results

### Inter-rater variability in odor pleasantness

Anise and chocolate were selected for their average neutral valence and the variability of pleasantness ratings they receive in the population. To verify that this was true in our sample, we examined both the average and individual ratings on the very first trial of each odor (i.e., at T1). As expected, anise and chocolate had moderate average pleasantness on the 0–100 hedonic scale, with large inter-individual variations (anise: *M* ± *SD* = 40.5 ± 25.2, range 1–100; chocolate *M* ± *SD* = 40.5 ± 25.2, range 1–85). The large variations in pleasantness ratings across participants allowed categorizing them as either “dislikers” or “likers” for each odorant. There were 14 “dislikers” (pleasantness ratings between 1 and 31 at T1) and 12 “likers” (ratings 47–100) for anise, and 13 “dislikers” (ratings 1–47) and 13 “likers” (ratings 50–85) for chocolate.

### Group analyses: “likers” and “dislikers”

**Figure 2:**
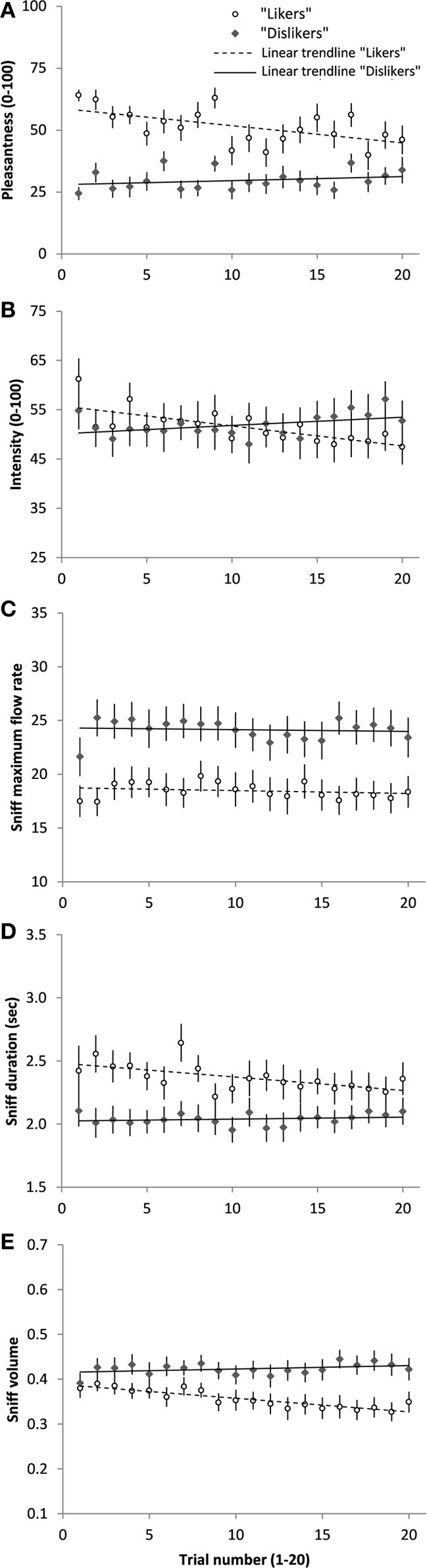
**Average hedonic ratings (A), intensity ratings (B), sniff maximum flow rate (C), sniff duration (D), sniff volume (E) (Mean ± s.e.m) of the “likers” and “dislikers” for the odors of anise and chocolate together, on each of the 20 repeated odor presentations.** The linear trend curve of each participant group is presented. Slopes and results of the linear regressions are shown in Table 1.

Average pleasantness, intensity, sniff maximum flow rate, sniff duration, and sniff volume of “likers” and “dislikers” across the 20 trials are shown in Figure 2. Results of the linear regressions between the five variables and time (Table 1) suggest that repeated exposure induced a significant decrease in pleasantness and intensity ratings, sniff duration, and sniff volume in “likers,” while these variables increased without reaching statistical significance in “dislikers.” In both groups, repeated-exposure resulted in a convergence of pleasantness ratings toward neutrality. Indeed, while hedonic ratings of “likers” and “dislikers” significantly differed at T1 (*t*-tests for independent samples, Table 1), they did not differ any more at T20. “Likers” and “dislikers” did not significantly differ on the other variables at T1 or T20, except for sniff maximum flow rate, higher in “dislikers” at T20 for chocolate. Finally, pleasantness ratings did not significantly correlate with intensity nor with sniffing parameters at T1 (Spearman rank correlations).

**Table 1:** **(A) Linear regressions between pleasantness, intensity, sniff maximum flow rate, sniff duration, sniff volume, and trial number (1 to 20) for “likers” and “dislikers” separately, and for the odors of anise and chocolate separately and together. (B) t-tests for independent samples between “likers” (L) and “dislikers” (D) at trial 1 and trial 20**.

	**(A) “Likers”**			**“Dislikers”**			**(B) Difference “Likers” vs. “Dislikers”**	
	**Slope**	***F*_(1, 18)_**	***p***	**Slope**	***F*_(1, 18)_**	***p***	***p* at T1**	***p* at T20**
**ANISE**								
Pleasantness	−0.37	1.11	0.306	+0.30	1.84	0.191	0.000^***^ L > D	0.577
Intensity	**−0.43**	**10.72**	0.004^**^	+0.14	1.17	0.294	0.544	0.272
Sniff max. flow rate	−0.03	0.54	0.471	−0.05	1.20	0.287	0.605	0.510
Sniff duration	**−0.011**	**4.59**	0.046^*^	+0.002	0.61	0.443	0.815	0.711
Sniff volume	**−0.003**	**37.36**	0.000^***^	+0.001	1.68	0.212	0.833	0.320
**CHOCOLATE**								
Pleasantness	**−0.99**	**14.45**	0.001^**^	+0.01	0.01	0.933	0.000^***^ L > D	0.127
Intensity	**−0.39**	**15.40**	0.000^***^	**+0.20**	**7.87**	0.012^*^	0.344	0.748
Sniff max. flow rate	−0.03	0.58	0.457	+0.02	0.19	0.670	0.074	0.026^*^ D > L
Sniff duration	**−0.012**	**7.12**	0.016^*^	+0.001	0.43	0.522	0.122	0.081
Sniff volume	**−0.003**	**22.59**	0.000^***^	+0.001	2.54	0.128	0.775	0.055
**BOTH ODORS**								
Pleasantness	**−0.69**	**9.01**	0.008^**^	+0.16	1.17	0.293	–	–
Intensity	**−0.40**	**20.07**	0.000^***^	+0.17	4.12	0.057	–	–
Sniff max. flow rate	−0.03	1.03	0.323	−0.02	0.21	0.650	–	–
Sniff duration	**−0.011**	**10.76**	0.004^**^	+0.002	0.86	0.367	–	–
Sniff volume	**−0.003**	**65.53**	0.000^***^	+0.001	2.71	0.117	–	–

*** p < 0.001;

** p < 0.01;

* p < 0.05.

### Correlation between individual initial pleasantness and time-related perceptual changes

**Figure 3:**
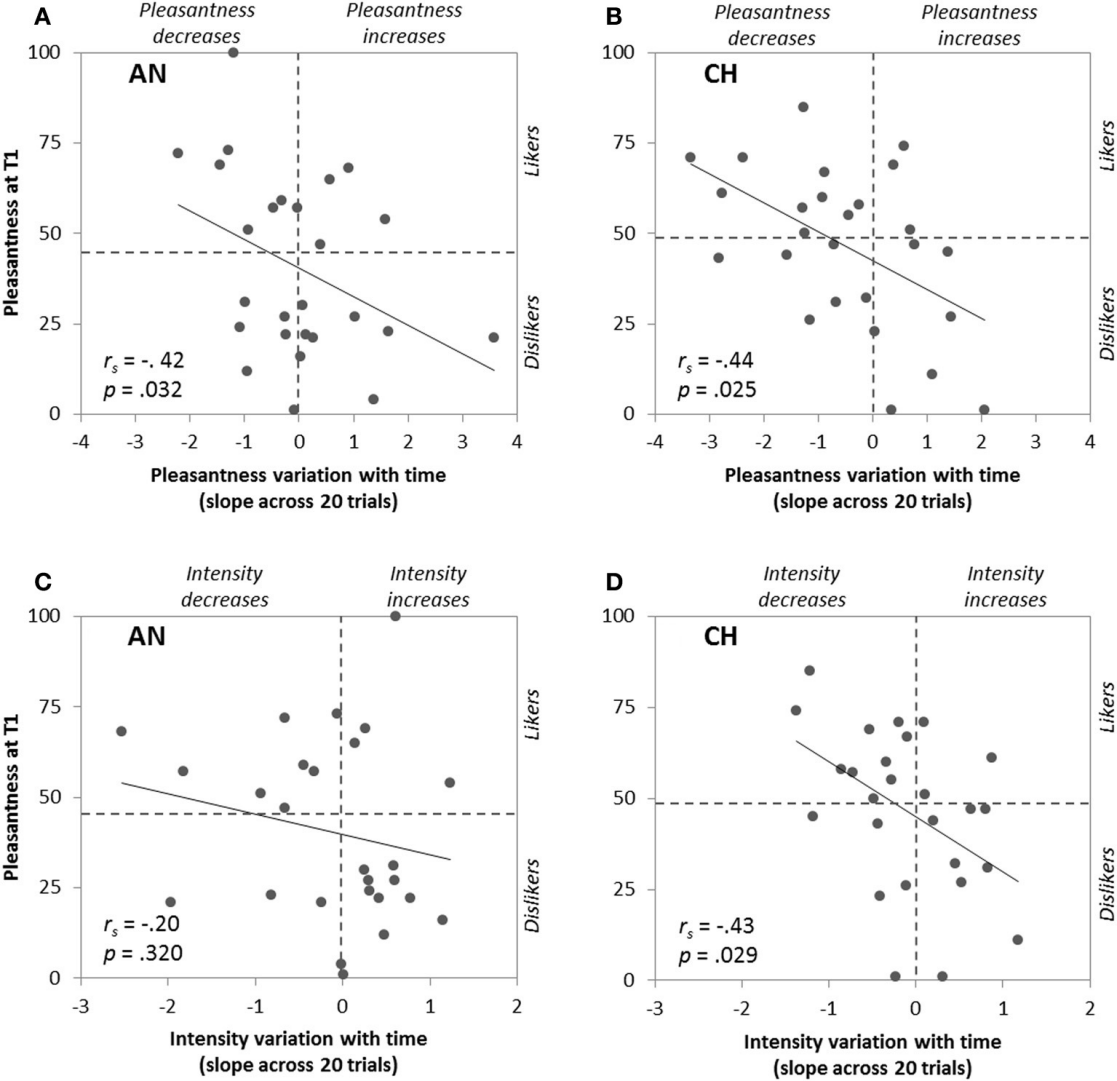
**Correlations between pleasantness ratings at T1 (first odor presentation) and time-related changes of pleasantness (A,B) and intensity (C,D) ratings represented by their slopes across the 20 odor presentations, for anise (AN) and chocolate (CH)**.

**Figure 4:**
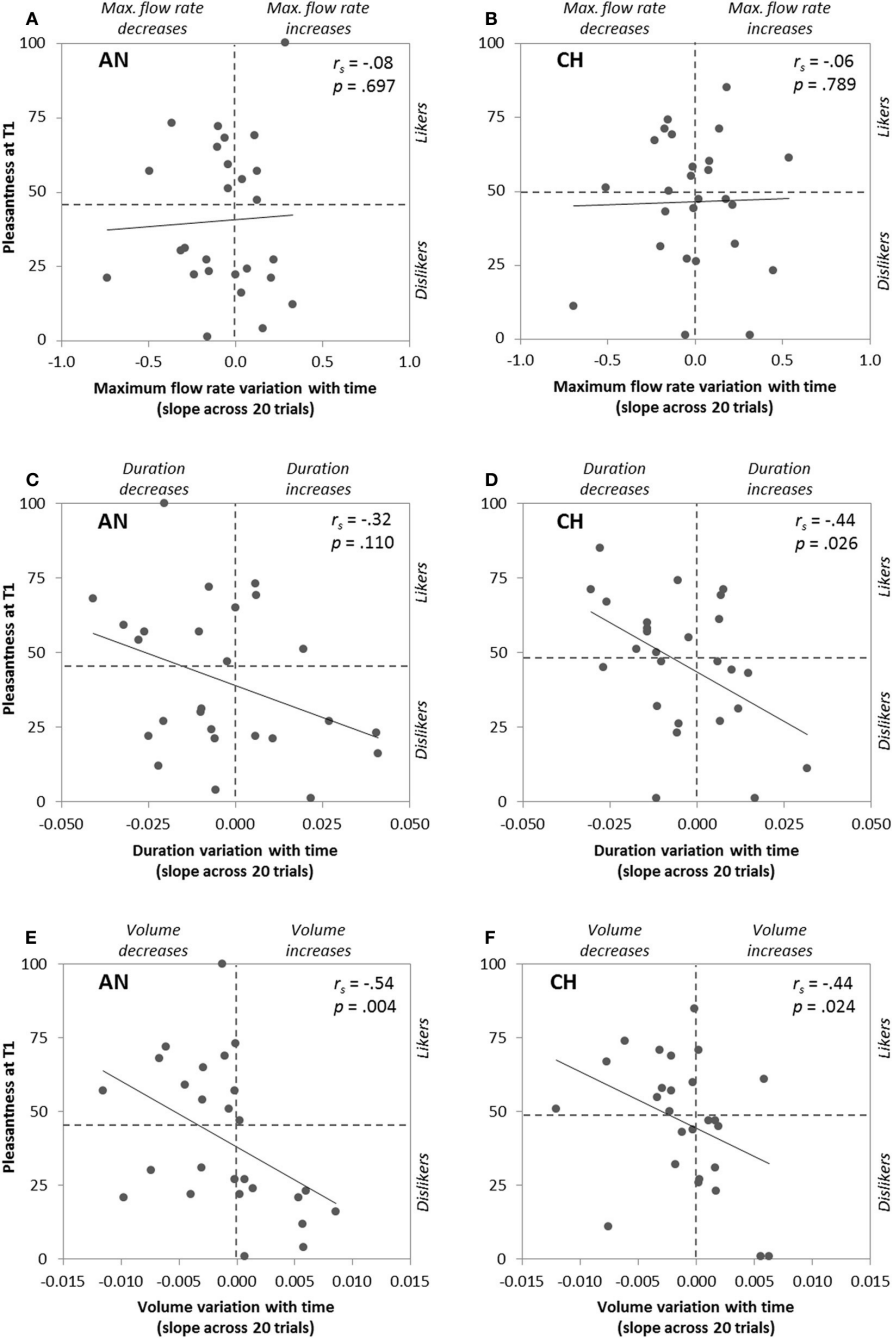
**Correlations between pleasantness ratings at T1 (first odor presentation) and time-related changes of the following sniff parameters: maximum flow rate (A,B), duration (C,D), and volume (E,F) represented by their slopes across the 20 odor presentations, for anise (AN) and chocolate (CH)**.

To go further, we then focused on each participant's pleasantness ratings at T1 and we correlated it with the time-related changes in pleasantness, intensity, and sniff parameters represented by the slopes of these variables as a function of trial number. The slopes were positive or negative depending on the participants (e.g., pleasantness ratings: range = −2.21 to +3.57, mean = 0.00 for anise, and range = −3.35 to +2.06, mean = −0.50 for chocolate). As expected, Spearman coefficients showed significant negative correlations between initial pleasantness and the slopes of the variables—except sniff maximum flow rate—for one or both odors. These results, illustrated in Figures 3, 4, mean that: (i) higher initial odor pleasantness ratings were associated with larger decreases of pleasantness, intensity, sniff volume and duration during repeated exposure (more negative slopes), and (ii) lower initial odor pleasantness ratings were associated with smaller decreases (slopes closer to zero) and even to increases of these variables (positive slopes), especially for the pleasantness ratings (Figures 3A,B) and the sniff volume (Figures 4E,F).

## Discussion

In the present study, we aimed at testing how hedonic perception of odors varies with repeated exposure, and whether inter-individual differences in hedonic perception of a given odor can modulate this variation. Namely, we used two odors people did not agree to find pleasant or unpleasant and presented them twenty times each (T1 to T20) in a random sequence. We explored time-related perceptual and motor (sniffing) changes for each odor, according to the participant's initial hedonic judgment. First, when considering the groups of “likers” (who rated the odor as pleasant at T1) and of “dislikers” (who rated the odor as unpleasant at T1), we found that pleasantness significantly decreased with time in “likers.” In “dislikers,” unpleasantness tended to decrease with time but the effect did not reach significance. These effects were paralleled by similar changes in intensity ratings, sniff duration, and sniff volume. We noticed that these effects led to a decrease in affective responsiveness since pleasantness ratings of both groups did not differ any more after 20 odor presentations. Second, when investigating more precisely the level of initial pleasantness rating at T1, we found negative correlations with the slopes (or time-related changes) of pleasantness, intensity, and sniff volume and duration for at least one odor. Correlation graphs (Figures 3, 4) show that higher initial pleasantness was mostly associated with more negative slopes (decrease in ratings and sniffing) and lower initial pleasantness was mostly associated with more positive slopes (increase in ratings and sniffing). In sum, we showed that affective habituation occurs with repeated exposure, which can be observed both at the self-reported level and at the olfactomotor level. We also provided evidence that repeated exposure influences individuals differently according to whether they initially liked or disliked the odor, affective habituation being more significant for odor “likers.”

One can wonder whether peripheral mechanisms such as olfactory adaptation may explain the present findings. Peripheral olfactory *adaptation* (or olfactory fatigue) is a phenomenon characterized by a decrease of the olfactory receptors' sensitivity due to prolonged or repeated exposure. Our experimental procedure was designed to limit such phenomenon by using appropriate inter-stimulus intervals (minimum 30 s) and by presenting two different odors randomly. Moreover, olfactory adaptation is characterized by a decrease in perceived intensity (Cain, 1969). Thus, if adaptation had occurred in our study, all participants should have displayed a decrease in perceived intensity, paralleled with an increase in sniff magnitude (see Laing, 1983; Frank et al., 2003, for the link between odor intensity and sniff volume/duration). However, this was not the case since a substantial number of participants displayed positive slopes over time for intensity, sniff volume and sniff duration (see Figures 3C,D, 4C–F). Rather, the time-related variation of pleasantness toward neutrality observed in our study is likely due to more central processes, and may therefore be preferentially qualified of *affective habituation*. It must be kept in mind that both processes are not independent (central processing can reflect changes in peripheral response) and the origin of response reduction due to repeated exposure remains unclear (Dalton, 2000).

Affective habituation is a form of learning that has been observed in previous studies, through decreasing strength of responses to repeated emotional stimuli of various nature, at the psychophysiological level (reduction of the electrodermal and electromyographic response: Bradley et al., 1993) and at the neurophysiological level (decrement in amydgala activation: Wright et al., 2001; Mutschler et al., 2010). At the behavioral level, few studies have described affective habituation using odors with contrasted pleasantness. Cain and Johnson (1978), who measured pleasantness of odors before and after repeated exposure, found a shift in the direction of hedonic neutrality: the positively valenced odor of citral became less pleasant and the negatively valenced odor of isobutyric acid became less unpleasant after exposure. Similarly, Prescott et al. (2008) showed an increase of pleasantness of two (neutral and unpleasant) odors after an exposure phase, as did Croy et al. (2013) after three presentations of the unpleasant odor of H_2_S. The latter result was corroborated by a reduced neuronal activation at the cerebral level and was interpreted as a decrease in emotional salience. With a more time-related approach, our study provided further evidence that this effect exists and is gradual: using a linear model of the pleasantness change across 20 odor presentations, we showed that pleasantness follows different trajectories, depending on the initial hedonic rating of the participants.

In this study, sniffing behaviors followed the same pattern as pleasantness ratings. This result reinforces the hypothesis that affective habituation occurs when an odor is repeated in a short period of time. Odor pleasantness is known to co-vary with sniffing behavior parameters, whether the odor is really smelled or whether it is imagined: compared to unpleasant odors (like rotten egg or fish), pleasant ones (like strawberry or rose) have been repeatedly found to be associated with larger and longer sniffs (Bensafi et al., 2003, 2007; Joussain et al., 2013). This motor correlate of odor pleasantness seems to be a robust mechanism since it is observed even when participants are asked to maintain constant sniffs across conditions (Bensafi et al., 2007). In line with this, we found that, as for pleasantness, sniff volume and duration mostly decreased over time in “likers” and tended to increase or stagnated in “dislikers.” In sum, not only did repeated exposure cause pleasantness to become more neutral, it also caused more involuntary parameters of olfactory perception (sniff duration and volume) to reflect this tendency toward neutrality. One may be surprised by the fact that “likers” and “dislikers” did not differ in their sniffing patterns for any of the two odors, and that pleasantness ratings did not correlate with sniffing volume or duration at T1. Relationship between sniff and pleasantness reported in the literature was usually found in response to odors with different qualities and more importantly, with highly contrasted valence (e.g., rose vs. rotten egg in Bensafi et al., 2003). In our study we compared individual responses to the same odor: not only are differences thus likely to be less marked but also inter-individual variability may have prevented the difference between “likers” and “dislikers” to reach statistical significance. Sniffing may nonetheless be considered as a reliable measure because, for a given individual, fine time-related changes paralleling changes in pleasantness were found in our study.

Why would affective habituation occur when odors are presented repeatedly? And how can this be interpreted in relation to another apparently contradictory theory, the mere exposure effect, according to which exposure leads to familiarization and higher liking (Zajonc, 1968)? In the conditions of our experimental design, namely 20 repeated presentations of two odors within about an hour, responsiveness to the repeated stimuli decreased. As nicely explained by Dijksterhuis and Smith (2002), habituation is a very useful mechanism that prevents us to be overwhelmed by the numerous stimulations of our environment. When encountering an emotional stimulus, such as an appetitive or a repulsive odor, we may first react intensely, but if subsequent repeated or prolonged exposure proves not to have any positive or negative consequence on the organism, such an intense response becomes unnecessary. On the course of time, the stimulus becomes less relevant, leading to reduced responsiveness. The effect of repeated exposure can be more pronounced or even reversed if the stimulus has effective or supposed consequences on the organism. For example, repeated chocolate ingestion, which has physiological outcomes, leads an initially very positive stimulus (chocolate) to lose its pleasantness (like in our study) and even to become aversive, and activates accordingly two different cerebral substrates related to reward and punishment, respectively (Small et al., 2001). Another example refers to unpleasant odors. If the odor were associated with the belief that it is harmful, by itself or via its source, responsiveness to the odor would then be more likely to increase rather than to decrease or remain stable like in our study. Indeed, in a study by Dalton (1996), perceived intensity of an odor increased over time for an odorous substance presented as being hazardous (sensitization), whereas it decreased in participants who believed that this substance was healthy. If pleasantness of the repeated harmful substance were measured, it probably would decrease over time (instead of increasing or remaining stable like in our study). These results highlight the importance of cognitive influences on odor perception, both at a given time (Herz and von Clef, 2001; De Araujo et al., 2005) and over time. The mere exposure effect, where novel (never encountered) stimuli that become more familiar with exposure also become more appreciated (Zajonc, 1968), may have the same origins as the habituation pattern of initially negative stimuli found in Cain and Johnson (1978) and more moderately in “dislikers” in our study. It is also the phenomenon that might occur in the case of cultural influences on odor perception: learning to associate initially negative smells with positive consequences (taste enjoyment of smelly cheese in France or of the foul-smelling durian fruit in Asia; Ayabe-Kanamura et al., 1998; Ferdenzi et al., 2013) may decrease its unpleasantness possibly to the point where it even reaches the positive side of the pleasantness scale.

In sum, repeated presentation of emotional stimuli such as odors may produce gradual decrease in responsiveness (tendency to neutral hedonic valence), but cognitive influences related to the consequences on the organism can modulate this pattern, by increasing responsiveness to repeated stimuli that have harmful or beneficial outcomes. In future studies, the asymmetry between affective habituation to pleasant and unpleasant odors (or of “likers” and “dislikers”) should be investigated further. Indeed, our study suggested that habituation was much less pronounced in “dislikers” than in “likers.” In “dislikers,” pleasantness and sniff magnitude seemed to have a tendency to increase with repeated exposure but the effect did not reach significance (Table 1), while reverse time-related changes were highly significant in “likers.” It might be that unpleasant odors are more resistant to the effect of familiarization, because maintaining an aversion for potentially harmful stimuli is an adaptive behavior (Delplanque et al., 2008; Ferdenzi et al., 2013). Affective habituation to unpleasant odors may thus be more limited in amplitude and/or might require longer exposure to reach the same magnitude as with pleasant stimuli, but this remains to be tested.

Finally, our study shows that it is highly relevant for olfaction studies to take into account inter-individual differences in hedonic perception. Agreement between raters and between cultures seems to be lower for neutral and pleasant odors than for unpleasant ones (Schaal et al., 1998). Hedonically neutral odors, in particular, may not be truly “neutral” and may rather receive highly contrasted odor ratings with some participants finding them pleasant and others finding them unpleasant (as in Doty, 1975), which leads to a moderate average score. Our study highlights significant differences from one person to another in the changes of perception and sniffing over time, for the same odor. When investigating odor hedonics, it is hazardous to consider the object *per se* independently of the perceiver (Robin et al., 1999; Rouby and Bensafi, 2002; Forestell and Mennella, 2005) because pleasantness is subjective and depends on personal past experience, current needs and goals.

## Author contributions

Moustafa Bensafi and Catherine Rouby designed the research, Camille Ferdenzi and Moustafa Bensafi analyzed the data and wrote the paper, Johan Poncelet conducted the data collection.

### Conflict of interest statement

The authors declare that the research was conducted in the absence of any commercial or financial relationships that could be construed as a potential conflict of interest.
